# A Retrospective Analysis of the Results of a Five-Year (2005–2009) Parasitological Examination for Common Intestinal Parasites from Bale-Robe Health Center, Robe Town, Southeastern Ethiopia

**DOI:** 10.5402/2013/694731

**Published:** 2013-12-11

**Authors:** Bayissa Chala

**Affiliations:** Department of Biology, School of Natural Sciences, Adama Science and Technology University, P.O. Box 1888, Adama, Ethiopia

## Abstract

A cross-sectional retrospective survey using the past five years clinical records (2005–2009) was conducted. The study was aimed at assessing the status of common intestinal parasites from Bale-Robe Health Center, Southeastern Ethiopia, in 2009/2010. The survey involved collection of data recorded on intestinal parasite from the health center during 2005–2009. Precoded questionnaires and interviews were also supplemented for knowledge attitude practices survey (KAPs survey) to assess awareness level of treatment seekers. Analysis of the various associations and strength of significant variations among qualitative and quantitative variables were assessed. The results revealed that *Entamoeba histolytica* (36.1%) and *Giardia lamblia* (11.0%), both being protozoan parasites were found to be the most prevalent intestinal parasites encountered during 2005–2009. The least prevalent intestinal parasite recorded was *Strongyloides stercoralis* (1.1%). Most intestinal parasites were detected among age group of 15 years and above than 0–4 and 5–14 years as shown in Table 4. There was a significant correlation between intestinal parasites prevalence and the age of treatment seeking individuals (*P* < 0.05). A sharp increasing trend of *E. histolytica* and *Ascaris lumbricoides* infections was observed owing to low personal and environmental sanitation of the majority of the society. Initiation of health education at different levels could be recommended to mitigate infectious parasites in the area.

## 1. Introduction

Parasitic infections are among the dominant contributors of morbidity and mortality and, hence, major public health problem worldwide. Many parasitic infections are associated with overcrowding, poor sanitation, contaminated food and water, undernutrition, and other poverty-related factors. Current estimates showed that at least more than one-quarter of the world's population is chronically infected with intestinal parasites and that most of these infected people live in developing countries [1–3]. Infections due to intestinal parasites are common throughout the tropics, posing serious public health problems in developing countries [4–6].

Intestinal parasitic infections, as in many developing counties, are common in Ethiopia and cause serious public health problems such as malnutrition, anaemia, and growth retardation as well as higher susceptibility to other infections [7].

Environmental factors play a central role in the transmission of intestinal infectious parasites in most rural African countries. The case of Bale-Robe may not be different, since there are enormous accesses by which these intestinal parasites can be transmitted. It is well known that eggs of some intestinal parasites like that of *Ascaris lumbricoides *and* Enterobius vermicularis *are carried by blowing dust particles as Bale-Robe appears to be highly favorable for such mode of transmission.

As far as sanitation of drinking water and food is concerned, there might not be pronounced care for personal and environmental hygiene in Bale-Robe town service providers such as cafeteria, restaurants, and hotels, as intestinal parasites can be contracted through contaminated food, water, and fomites.

Improved sanitation which mainly includes avoidance of food and water contamination and health education about the modes of transmission of infections are the two key components of preventive and control measures for intestinal parasites [8]. The survey on prevalence of intestinal parasites provides information concerning the burden of parasites in a community, enabling to predict risks for communities under consideration and decisions to be made as to the need for intervention [9].

In spite of the tremendous prevalence of intestinal parasites which can be guessed from the prevailing facilitating favorable conditions, no study of any sort regarding intestinal parasites has so far been conducted in Bale zone including Robe town. On the other hand, study on nutrition survey in Goro woreda focus group discussions revealed that the perception of community members related to causes of malnutrition and sickness in young children focused mainly on use of unclean water collected from unprotected sites such as depleted rivers and ponds, out of necessity [10]. In order to investigate the prevalence of common intestinal parasites with the aforementioned perspectives, this study was initiated.

## 2. Methods

The study was conducted in Bale-Robe town which is one of the recently growing towns in Oromia Regional State located at 430 Km southeast of the capital of Ethiopia. The town has a moderately highland climate with average minimum and maximum temperature ranges of 9.42°C and 21.16°C, respectively. The average minimum and maximum annual rainfall are 535 mm and 1018 mm, respectively [11]. The climate is seemingly conducive for the survival and development of parasites.

 A retrospective record review based on data of the past 5 years was used in this cross-sectional survey. The data on outpatient intestinal parasitic cases for 2005–2009 years was collected from Robe town Health Center. The stool specimens were examined using different parasitological analysis including direct smear and 10% formalin concentration method [12, 13]. The age, sex, and other demographic information of the patients were recorded. Data of intestinal parasites collected from the health center was properly recorded and filed. Furthermore, observational study and questionnaire were implemented to strengthen the study (Table 2).

Data collection was followed by proper data organization, analysis, and interpretation. Retrospective data obtained from the health center and data from questionnaire were qualitatively and quantitatively analyzed to check the frequency and strength of associations of variables. Results were expressed as rates and proportions. Chi-square test of statistical significance was applied to study the association between prevalence of intestinal parasites and the demographic factors. *P* value < 0.05 was considered as significant.

## 3. Results

The present retrospective study showed that the prevalence of intestinal parasite infection during 5 years ranged from 0.3% to 36.1%. There were nine different parasites encountered. The most common intestinal parasites identified were *Entamoeba histolytica* (16.0%–36.1%) and *Giardia lamblia* (6.1%–11.0%) as clearly presented in Table 1. *Strongyloides stercoralis *showed the least prevalence (0.9%–1.8%). The other parasites present were* Ascaris lumbricoides, Hymenolepis nana*,* Enterobius vermicularis*,* Taenia *species, and Hookworms (Table 1).

**Figure 1:**
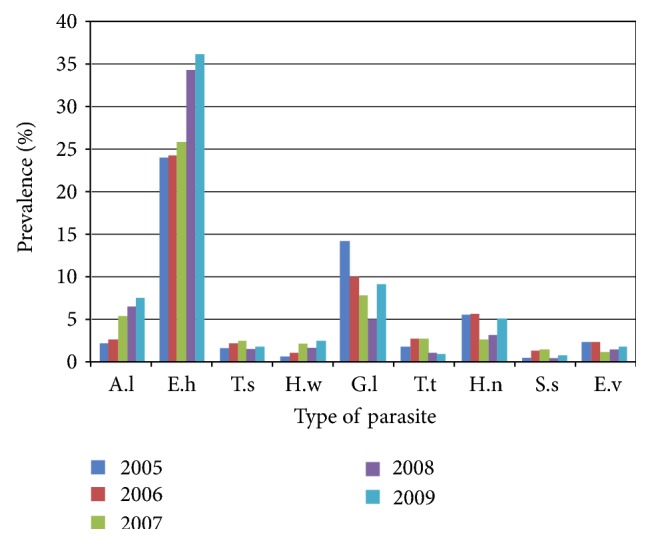
Comparison of prevalence of intestinal parasite from Bale-Robe Health Center, 2005–2009. Abbreviations: A.l: *Ascaris lumbricoides*; E.h: *Entamoeba histolytica*; T.s: *Taenia* species; H.w: hookworm; G.l: *Giardia lamblia*; T.t: *Trichuris trichiura*; H.n: *Hymenolepis nana*; S.s: *Strongyloides stercoralis*; E.v: *Enterobius vermicularis*.

*Entamoeba histolytica *was found to be the most prevalent with average prevalence of about 26.5% during the 5 years (2005–2009). And *Giardia lamblia* was found to be the second most prevalent peaking up to 11.0% and with average prevalence of 8.5% during the same period, but by 2008, it was exceeded by *Ascaris lumbricoides* (6.8%). Clinical data of the past 5 years from Bale-Robe Health Center were presented in Figure 1.

## 4. Knowledge Attitude Practices Surveys (KAPs Surveys)

Out of 45 respondents, 15 (33%) male and 16 (35.6%) female respondents reported that they have been provided general health care education at their residents. Similarly, 15 (33.3%) male and 8 (17.8%) female were found to have been educated at Bale-Robe Health Center. All respondents replied that they have toilet and also wash their hand after visiting latrine. 

Regarding the exposure of the treatment seeking person to common intestinal parasites, 3 (6.7%) male and 2 (4.4%) female respondents indicated as they experienced intestinal parasites at least once in their life, and the previous respondents who experienced intestinal parasite said that they prefer visiting health facility immediately after they feel intestinal discomfort.

On the other hand, when respondents were asked about modes of transmission of intestinal parasites, the majority (90%) replied that food and water contamination would be the major route of transmission. Others responded that lack of personal hygiene and environmental hygiene could be the major route of transmission. Insignificant number of respondents (5%) responded that air pollution could be optional mode of transmission for intestinal parasites. Similarly, very few (<5%) respondents replied that they do not know the way of transmission of intestinal parasites.

Concerning treatment site, the vast majority of the respondents 41 (91%) replied that they prefer visiting modern treatment organization than traditional medical center. The reasons for visiting modern health medication over traditional treatment centers as replied by most respondents include lack of appropriate dose, insufficient knowledge about a drug, lack of appropriate investigation on the drug, and harmful side effects of the prescribed drug regimens in the later. Only insignificant number of respondents (<10%) replied that they can use both modern and traditional treatment method primarily because of the lack of health facilities near by their residence.

The age of Bale-Robe Health Center staff respondents ranged from 23 to 54 years with 38.5 average. And 60% of the health center staff respondents were males and 40% were females (Table 3).

Regarding general health education service provision at the health center, all responded that there has been daily activity of educating of the treatment seekers. The staff respondents were also asked whether or not the health center has adequate skilled human power in each health service provision departments, about 70% replied yes. On the contrary, about 70% of the respondents replied that the health center laboratory setup is not equipped with necessary equipment and chemical reagent. Concerning the occurrence of intestinal parasites among the examined patients at the health center, 100% of the staff respondents agreed as there was usual encounter of common intestinal parasites.

Similarly, *Entamoeba histolytica*, *Ascaris lumbricoides*, *Taenia* species, hookworms, and *Giardia lamblia* were found to be among the most frequently encountered intestinal parasites as replied by the staff respondents. In relation to the reason stated for the frequent occurrence of the aforementioned intestinal parasites, most staff respondents associated with lack of personal hygiene and environmental sanitation as well as poor awareness towards the preventive measures.

## 5. Discussion

Intestinal parasitic infections are among the most common infections worldwide. It is estimated that some 3.5 billion people are affected, and 450 million are ill as a result of these infections [14]. The rate of infection is remarkably high in Sub-Saharan Africa, where the majority of HIV/AIDS cases are concentrated [15]. The incidence of intestinal parasitic infections is 50% in developed countries, whereas it reaches up to 95% in developing countries. These infections are caused both by protozoa and helminthes, with main clinical manifestation of diarrhoea [16].

In the present retrospective study, nine parasites were identified with an overall average prevalence of 6.23%. A retrospective study conducted on intestinal parasites from St. Lucia, a Caribbean island, revealed an average prevalence of 26.1% (24.5%–27.7%) [17]. As reported in the prospective study conducted among Kara and Kwego semipastoralist tribes in lower Omo Valley, nine parasites with prevalence of 104 (51.7%) and 83 (41.5%) were identified, respectively [18]. More recently, a study conducted in Diga district, East Wollega zone, showed an overall prevalence of 64.9% intestinal parasites in general and 49.7% hookworm prevalence in particular in the area [19]. Similarly, retrospective study in Palestine showed that the prevalence of intestinal parasite infection during 10 years (2000–2009) ranged from 32.0% to 41.5% with *Entamoeba histolytica* (8.2%–18.2%) and *Enterobius vermicularis* (15.6%–28.9%) [20].

Since the transmission of *E. histolytica* (36.1%) and *G. lamblia* (11.0%) is feco-oral route, higher prevalence of the parasites may be associated with poor personal and environmental hygiene. This higher prevalence of *E. histolytica *and* G. lamblia *goes with the report of WHO report which pointed out these two parasites as common causes of intestinal infection throughout Ethiopia. Moreover, it has been indicated that amoebiasis and ascariasis are among the ten most infections globally [21]. It has been estimated that about 200 million people are infected each year in Africa, Asia, and Latin America by giardiasis [22]. It was reported that the prevalence of giardiasis among children in relation to water sources in a selected village of Pawi Special District in Benishangul-Gumuz Region, Northwestern Ethiopia, was 26.6% [23].

In the present retrospective study, *Ascaris lumbricoides* with 6.8% average prevalence and *Hymenolepis nana* (4.3%) exhibited the third and fourth highest infection rate following *E. histolytica* and *G. lamblia* during most seasons of 2005 through 2009 (Table 1). As reported by [21], the average prevalence of ascariasis in most African countries was 32% ranging from 16% to 48%. Similar to the two protozoa parasites mentioned earlier, *H. nana *and* A. lumbricoides *spread through feco-oral route implying that their higher prevalence can be correlated to poor personal and environmental hygiene.

Hookworms were among the average prevalent intestinal parasites with prevalence ranging from 2.1% to 3.8% throughout the years 2005–2009. On the whole, *S. stercoralis* exhibited the least average prevalence (1.1%) as shown in Table 1. Less prevalence of *S. stercoralis* infection could be associated with multiple factors. For instance, presence of latrine plays a central role in preventing the access of larvae to human beings. This is because the mode of transmission of *S. stercoralis* as well as hookworms is mainly occurred by active penetration of the larvae through human skin. In the same way, wearing shoe by most people and pure water supply may contribute a lot for the less prevalence of the indicated parasite. Moreover, it is usual to use tractor on farm lands and spraying herbicides by Robe town surrounding farmers which may reduce direct contact of the larvae in the soil to human skin.

Regarding distribution of the intestinal parasites among age groups, more infection rate was observed in the age of 15 years and above through the years 2005 to 2009 followed by age groups 5–14 and 0–4 years as presented in Table 4. Studies have found a strong correlation between increasing age and increasing prevalence and intensity of hookworm infection [24–27].

With regard to the sex distribution of hookworms and *S. stercoralis*, they were found to be more prevalent in males than females throughout 2005–2009 as can be observed in Table 1. Such deviation of these two parasites towards males other than females appears to be associated with occupation of individuals. In India, the findings of a study on the risk of intestinal nematode (hookworm infections) revealed high risk in farming families occupationally exposed to untreated and partially treated wastewater [28], while the other parasite showed no significant correlation with either sex of the individuals. On the other hand, studies have found out that *H. nana* predominantly affected males morethan females among dwellers of Jimma town [29]. However, in the present study, there is no much variation in relation to the sex of individuals.

In particular, *Taenia* species, whose transmission is associated with eating beef or pork, were found to be more common among adult age groups. This can be explained in terms of cultures of the society in which raw meat is more commonly consumed by adults than children. Research reports from Jimma town showed that parasites that are transmitted via contaminated hands or feco-oral route like *E. histolytica*, *G. lamblia,* and *H. nana* possessed high prevalence in children especially in those under 5 years than adults [29]. This could be associated with the development of immunity and better awareness in washing hands and personal hygiene in adults. But the present study revealed that *E. histolytica* and *G. lamblia* showed higher prevalence in adults. This may be due to lack of relevant information, due to higher interpersonal interaction by going away from one's own area, feeding of raw or undercooked vegetables and improperly washed vegetables.

With regard to the evaluation of the past 5 years status of common intestinal parasites detected at the health center from Knowledge Attitude Practices survey, about 70% of the staff respondents agreed on decreasing trend. The reasons behind the decline of intestinal parasites in the past 5 years as indicated by the respondents included the provision of health education at different sites (60%) and the supply of pure water (10%). The rest 20% replied that there was an increasing trend of the common intestinal parasites due to poor awareness of the society towards preventive measures and poor personal and environmental hygiene. Only 10% of the staff respondents replied that there was constant trend of intestinal parasites at the health center. However, retrospective data of the present study showed mixed figure, that is, there was a sharp escalating trend for some intestinal parasite like *E. histolytica *and* A. lumbricoides *through the past five years (2005–2009). On the other hand, the rest of parasites showed variable trend of distribution.

Regarding the possibility of control of common intestinal parasites in relation to the prevailing condition of Robe town and the awareness level of the society, about 60% of the respondents replied that it is impossible to control. This is because the environmental conditions (like runoff, blowing dust, etc.) are favorable for the transmission of the parasites. It is a well-established idea that eggs of some intestinal parasites such as *Ascaris lumbricoides *and* Enterobius vermicularis *are carried by blowing dust particles and transmitted by inhalation [30]. On the other hand, 40% of the staff respondents agreed on the possibility of control of the parasites due to the presence of clean water supply and environmental sanitation. There was a significant correlation between intestinal parasites prevalence and the age of treatment seeking individuals (*P* < 0.05).

## 6. Conclusions

Based on the results of this piece of work the following conclusions were drawn.

1. *Entamoeba histolytica* and *Giardia lamblia* were found to be the most prevalent intestinal parasites among individuals visited the health center during the past five years (2005–2009).
2. Although retrospective data of the present study showed mixed figure for the prevalence of most of the intestinal parasites, there was a sharp increasing trend for some intestinal parasites like *E. histolytica* and *Ascaris lumbricoides* throughout the past five years (2005–2009).
3. Most intestinal parasites were more prevalent among individuals 15 and above years old than individuals less than 15 years.

## Figures and Tables

**Table 1:** Prevalence (in percent) of intestinal parasites stratified by year and host gender (*n* = number infected), 2010.

	2005			2006			2007			2008			2009			Total sample
Parasite	No. (%)			No. (%)			No. (%)			No. (%)			No. (%)			
	M	F	T	M	F	T	M	F	T	M	F	T	M	F	T
*A. lumbricoides *	18 (3.7)	28 (5.5)	46 (4.6)	24 (4.2)	17 (3.0)	41 (3.6)	31 (6.3)	24 (4.5)	55 (5.3)	73 (11.6)	58 (7.1)	131 (9.0)	93 (12.2)	65 (6.6)	158 (9.0)	431 (6.8)
*E. histolytica *	76 (15.6)	84 (16.3)	160 (16.0)	103 (18.0)	92 (16.7)	195 (17.3)	136 (27.7)	128 (23.9)	264 (25.7)	186 (29.5)	246 (30.2)	432 (29.9)	324 (42.6)	308 (31.1)	632 (36.1)	1683 (26.5)
*T. *species	11 (2.2)	13 (2.5)	24 (2.4)	12 (2.1)	17 (3.1)	29 (2.6)	8 (1.6)	16 (3.0)	24 (2.3)	17 (2.7)	13 (1.6)	30 (2.1)	27 (3.6)	13 (1.3)	40 (2.3)	147 (2.3)
Hookworm	11 (2.1)	10 (2.1)	21 (2.1)	14 (2.4)	10 (1.8)	24 (2.1)	16 (3.2)	6 (1.1%)	22 (2.1)	26 (4.1)	18 (2.2)	44 (3.0)	36 (4.7)	28 (2.8)	66 (3.8)	177 (2.8)
*G. lamblia *	31 (6.4)	33 (6.4)	64 (6.4)	32 (5.6)	37 (6.7)	69 (6.1)	47 (9.6)	34(6.3%)	81 (7.9)	92 (14.6)	67 (8.2)	159 (11.0)	92 (12.1)	78 (7.9)	170 (9.7)	543 (8.5)
*T. trichiura *	5 (1.0)	7 (1.3)	12 (1.2)	7 (1.2)	9 (1.6)	16 (1.4)	15 (3.1)	13 (2.4)	28 (2.7)	7 (1.1)	8 (0.9)	15 (1.0)	11 (1.4)	9 (0.9)	20 (1.1)	91 (1.4)
*H. nana *	13 (2.7)	15 (2.9)	28 (2.8)	17 (3.0)	15 (2.7)	32 (2.8)	25 (5.1)	12 (2.2)	37 (3.6)	41 (6.5)	47 (5.8)	88 (6.1)	47 (6.2)	45 (4.5)	92 (5.3)	277 (4.3)
*S. stercoralis *	7 (1.3)	5 (1.0)	12 (1.2)	7 (1.2)	4 (0.7)	11 (1.0)	11 (2.0)	6 (1.2)	17 (1.6)	8 (1.2)	6 (0.7)	14 (0.9)	11 (1.4)	8 (0.8)	19 (1.8)	73 (1.1)
*E. vermicularis *	7 (1.4)	9 (1.7)	16 (1.6)	8 (1.4)	11 (2.0)	19 (1.7%)	9 (1.8)	12 (2.2)	21 (2.0)	15 (2.4)	14 (1.7)	29 (2.0)	15 (2.0)	17 (1.7)	32 (1.8)	117 (1.8)
Sample size (*n*)	485	513	998	572	552	1124	490	535	1025	630	815	1445	760	990	1750	6342

**Table 2:** Demographic information of treatment seeking respondents visiting Robe Health Center, 2010.

Sex	Age (in years)		Occupation					Total
	10–24	Above 25	Student	Housewife	Farmer	Trader	Government employee
Male	12	13	5	—	7	9	4	25
	11	9	—	8	—	3	5	20

Total	23	22	5	8	7	12	9	45

**Table 3:** Demographic information of Robe Health Center staff respondents, 2010.

Sex	Position				Total
	Health officer	Lab. tech.	Nurse	Pharmacy tech.
Male	1	2	2	1	6
Female	1	—	3	—	4

Total	2	2	5	1	10

**Table 4:** Proportion of parasites detected at Robe Health Center based on age ranges from 2005 to 2009.

	Age ranges (in years)								
Parasite	0–4			5–14			≥15		
	No. (%)			No. (%)			No. (%)		
	M	F	T	M	F	T	M	F	T
*A. lumbricoides *	45 (0.71)	37 (0.6)	82	63 (1.0)	52 (0.82)	115	131 (2.1)	103 (1.6)	234
*E. histolytica *	272 (4.3)	160 (2.5)	432	298 (4.7)	210 (3.3)	508	515 (8.1)	428 (6.8)	943
*T. species *	12 (0.19)	11 (0.17)	23	20 (0.32)	16 (0.25)	36	43 (0.68)	45 (0.71)	88
Hookworm	21 (0.33)	8 (0.12)	29	26 (0.41)	17 (0.27)	43	55 (0.87)	48 (0.76)	103
*G. lamblia *	64 (1.0)	7 (0.11)	71	85 (1.3)	12 (0.19)	97	145 (2.3)	26 (0.41)	171
*T. trichiura *	6 (0.09)	8 (0.12)	14	13 (0.2)	14 (0.22)	27	26 (0.41)	24 (0.38)	50
*H. nana *	22 (0.35)	28 (0.44)	50	31 (0.49)	35 (0.55)	66	90 (1.4)	71 (1.11)	161
*S. stercoralis *	5 (0.08)	6 (0.09)	11	8 (0.12)	9 (0.14)	17	21 (0.33)	24 (0.38)	45
*E. vermicularis *	10 (0.16)	15 (0.24)	25	13 (0.2)	20 (0.32)	33	31 (0.49)	28 (0.44)	59

Total sample					6342				
